# Effectiveness of Regional Nerve Blocks Versus Local Anesthetic Infiltration for Elective Hand and Wrist Surgery

**DOI:** 10.7759/cureus.63569

**Published:** 2024-07-01

**Authors:** Wahid M Hassan, Hivi Mahmoud

**Affiliations:** 1 Surgery, University of Duhok, Duhok, IRQ; 2 Medical Chemistry, University of Duhok, Duhok, IRQ

**Keywords:** ultrasound guided, pain management, hand and wrist surgery, local anaesthesia, nerve block

## Abstract

Background: Pain relief by different methods in elective hand and wrist surgery is pivotal for patients undergoing elective wrist and hand surgery.

Aim: To evaluate the effectiveness and duration of peripheral nerve block versus local surgical site anesthetic infiltration among patients undergoing elective wrist and hand surgery.

Subjects and methods: This study was carried out in the Orthopaedic Department of Duhok Emergency Teaching Hospital, Duhok, Kurdistan region, Iraq. All patients who attended the Orthopaedic Department (total number=496) for elective hand and wrist surgery between November 2021 and November 2022 were included in the study, but only 300 patients completed the study protocol after the exclusion of 196 patients. Three methods of nerve block were used for postoperative pain relief. The degree and duration of pain relief were assessed.

Results: Patients who underwent ultrasound-guided nerve block had more duration of pain relief when compared to those patients who underwent local surgical site anesthetic infiltration and anatomical landmark nerve block (p<0.01), while patients who underwent local surgical site anesthetic infiltration had better pain relief when compared to those patients who underwent ultrasound-guided nerve block and anatomical landmark nerve block (p<0.01 for the group experiencing mild pain and p=0.12 for the group experiencing moderate pain and p<0.01 for the group experiencing severe pain).

Conclusion: Ultrasound-guided nerve block is better for a longer duration of pain relief postoperatively than local surgical site anesthetic infiltration, while local surgical site anesthetic infiltration more effectively controls the severity of pain following elective hand and wrist surgery.

## Introduction

Nerve block for wrist and hand surgery is a valuable anesthetic method for pain control postoperatively, decreased recovery time from an operation, and early discharge of the patient from the hospital [1]. Currently, this nerve block is known to be more effective when performed with ultrasound guidance as the perineural injection technique [2]. However, this technique carries risks of direct nerve injury by the needle due to limitations of US imaging related to inter-individual anatomical variation and operator-dependent skills. It also sometimes requires repositioning of the needle, which can increase the risk of vessel damage and other complications [3].

Several previous trials showed comparable success rates in achieving pain control and shortening the post-operative remaining time in hospital [4-6]. There are many methods and techniques used for pain relief following surgery; they include analgesia, local surgical site infiltration, and nerve blocks. Local surgical site anesthetic injection has been commonly used after hand and foot surgery [7-9].

For better controlling of the pain postoperatively, it is essential to know the relationships between body surface anatomical landmarks and internal position of the peripheral nerves in order to have a safer surgery in addition to better analgesia [10].

The peripheral nerve block with anesthetic substance injection is better performed using an ultrasound guide; however, its effectiveness is dependent on ultrasound availability and user ability skills and should not replace the knowledge of the anatomy of the surgeon [11].

Because little data are known in our locality regarding the uses of different methods for pain relief postoperatively, this guides us to do this study to evaluate the effectiveness and duration of peripheral nerve block versus local surgical site anesthetic infiltration among patients undergoing hand and wrist surgery in Duhok, Kurdistan region, Iraq.

## Materials and methods

This study was carried out in the Orthopedic Department of the Duhok Emergency Teaching Hospital, Duhok, Kurdistan region, Iraq. All patients aged 18-75 years old who attended the Orthopedic Department (total number=496) between November 2021 and November 2022 and met the inclusion criteria were included in this study. After the exclusion of 196 patients who were not eligible for the study criteria and some of them did not agree to take part in the study, 300 patients completely met the study criteria and were included in this study. Three methods of postoperative pain control were selected randomly for patients who were divided into three groups (each group number=100). These methods of nerve block were the following.

Ultrasound-Guided Nerve Block

With the aid of a radiology specialist, all equipment needed was provided including an ultrasound machine, sterile sleeve, and gel as well as a 22-25 gauge (20 mL) syringe. Under ultrasound guidance, we place the needle tip immediately adjacent to each of the two/three nerves and then infiltrate 10-15 mL of 1% lidocaine with no epinephrine to deposit local anesthetic until it spread around the nerve [12].

Anatomical Landmark Nerve Block

The radial nerve block was performed with 5 mL of 1% lidocaine injected subcutaneously just above the radial styloid while advancing the needle medially. The infiltration is then extended laterally, using an additional 5 mL. An ulnar nerve block is done by inserting the needle under the tendon of the flexor carpi ulnaris muscle close to its distal attachment just above the styloid process of the ulna. The needle is advanced 5-10 mm to just past the tendon of the flexor carpi ulnaris. Additionally, 3-5 mL of 1% lidocaine solution was injected, and then an additional 2-3 mL was injected subcutaneously just above the tendon of the flexor carpi ulnaris to block the cutaneous branches of the ulnar nerve. Furthermore, a median nerve block was performed by inserting the needle between the tendons of the palmaris longus and flexor carpi radialis until it contacts the bone. At that point, the needle was withdrawn 2-3 mm, and then 3-5 mL of 1% lidocaine was injected [13].

Local Surgical Site Infiltration

Under direct visualization, we infiltrated 5-15 mL of 1% lidocaine with no epinephrine into all the wound layers before the wound closure [14].

These methods of postoperative pain control were clarified and explained to all participants, and written consent was obtained from each one to take part in the study. Inclusion criteria for patients studied included carpal tunnel syndrome release, trigger thumb and finger release, all hand and finger non-emergency injuries, tendon surgeries in the hand, and ganglion surgeries. Exclusion criteria were patients below 18 years old and those who have diabetes mellitus, who smoke, who have malignancy, who underwent chemotherapy and hand and wrist surgeries, who have a history and/or recent nerve injury, and who were cerebral palsy and stroke patients. The degree and duration of pain relief were evaluated by an anesthesiologist according to the quality of anesthesia graded by anesthesiologist (QAGA) [15]. Patients were grouped according to the duration of pain relief in the postoperative period: less than 10 minutes, 11-15 minutes, 16-20 minutes, 21-30 minutes, 31-35 minutes, 36-40 minutes, and 41-45 minutes. According to the severity of pain relief, which was performed using a visual pain analog [16], patients were graded into those with mild pain (0-3), moderate pain (4-6), and severe pain (7-10) groups. The study protocol was approved by the Committee of Medical Ethics in the Duhok Directorate of Health.

Statistical analysis

The number and percentage (n & %) were used in tables to adopt data of different groups, and the number and percentage (n & %) of data were plotted by clustered columns regarding the duration and severity of pain relief among groups with different techniques. The chi-square test was used to assess the degree of statistical significance between groups. A statistical cutoff value was set at a p value equal to or less than 0.05 (p<0.05).

## Results

Table 1 shows the percentage of patients included in the study regarding the duration of pain relief using different techniques. Fifty patients (50%) experienced pain relief less than 10 minutes when using anatomical landmark nerve block, and 51% of patients experienced pain relief less than 10 minutes when using intraoperative local surgical site infiltration, while only 13% had pain relief less than 10 minutes when using an ultrasound-guided nerve block. Twenty percent of patients experienced pain relief for more than 35 minutes when using an ultrasound-guided nerve block, while no patients (0%) experienced pain relief for more than 35 minutes when using intraoperative local infiltration and anatomical landmark nerve block.

**Table 1:** Duration of pain relief among groups with different techniques *Comparing USG nerve block vs intraoperative local infiltration for >20 minutes

Anaesthesia time	Intraoperative local infiltration (N=100) (%)	USG nerve block (N=100) (%)	ALM block (N=100) (%)	P value
<10 minutes	51	13	50	<0.01*
11-15 minutes	34	13	25	
16-20 minutes	8	14	25	
21-30 minutes	5	20	0	
31-35 minutes	2	20	0	
36-40 minutes	0	7	0	
41-45 minutes	0	13	0	

**Figure 1:**
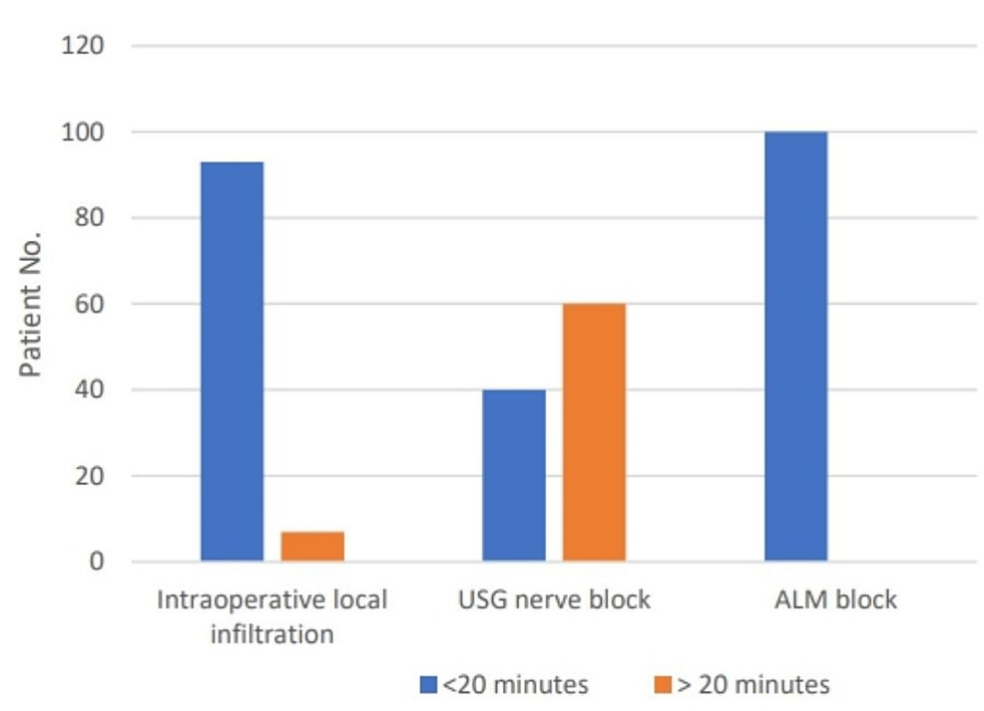
Duration of pain relief among groups with different techniques

Duration of pain relief of more than 20 minutes was seen in 60% when using ultrasound-guided nerve block, while only 7% of patients had pain relief of more than 20 minutes when using intraoperative local infiltration, and the difference was statistically significant (p<0.01). However, all patients (100%) experienced pain relief in less than 20 minutes when using an anatomical landmark nerve block (Figure 1).

**Figure 2:**
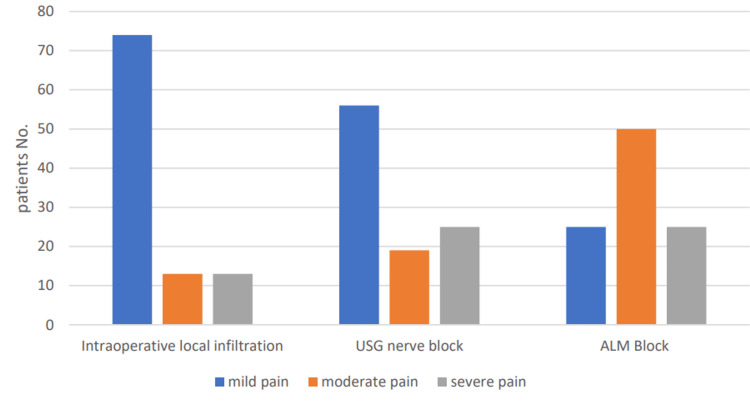
Percentage of pain relief among groups with different techniques

Approximately 74 patients (74%) had mild pain postoperatively when using intraoperative local infiltration compared to 56% for those using USG nerve block (p<0.01) and 25% for those using anatomical landmark nerve block (p<0.01) (Figure 2).

## Discussion

There are many procedures performed for hand surgery everywhere, but only a few researches concentrate on pain control postoperatively. Data regarding this are largely controversial, and, as such, there is no consensus on which treatment regimen is the most appropriate one. Surgical site infiltration, which is also known as local infiltration analgesia, is easy to perform, safe, and inexpensive. Additionally, it avoids motor blockade [14], while using anatomical landmark nerve blocks needs more precise and expert personnel [13] and ultrasound-guided nerve blocks need the assistance of an expert radiologist [12]. The results of pain control and its duration of action are controversial among these three methods, which interested us to do this study, to compare the results of these three methods of a nerve block to each other and other studies' results. Interventions for pain control differ from easy ones with the administration of oral painkillers to infusion treatment. The long duration of pain relief of more than 20 minutes was found among patients who underwent elective hand and wrist surgery when we used ultrasound-guided nerve block, and it was noticed in more than half of all patients included (60%). This finding was similar to another study done by Johnson et al. who showed longer pain control duration [17]. This relatively long duration of pain relief may be attributed to a more precise distribution of anesthetic substances around the nerve.

Another interesting finding of this study was the severity of pain relief. We found that 74% of the patients included had mild pain postoperatively when using local surgical site infiltration with an anesthetic substance for pain relief compared to 56% and 25% in those using ultrasound-guided nerve block and anatomical landmark nerve block, respectively. These findings disagree with another study by Bao et al. who found better pain relief when using an ultrasound-guided nerve block for hand surgery [18].

Our study, in combination with the previous studies [17-18], suggests that more pain relief in local surgical site infiltration is because of the precise localization of anesthetic substances in the field of surgery around the nerve endings that cause nerve blocks, but possible irrigation and/or absorption of the anesthetic substance may cause pain experience to come back sooner. However, a longer duration of pain relief using ultrasound-guided nerve block may be attributed to nerve block out of the surgical field, which is neither irrigated nor absorbed rapidly.

Limitations of the study

The use of one type of local anesthetic substance and the small sample size were the two main limitations of this study.

## Conclusions

The present study confirmed the fact that using an ultrasound-guided nerve block is better for a longer duration of pain relief than other methods used, while local surgical site infiltration is more effective in pain relief following elective hand and wrist surgery. More studies with larger sample sizes and more follow-up periods using more than one method for pain control are recommended to have more trustworthy results for postoperative pain control in elective hand and wrist surgeries.
